# Tissue-type plasminogen activator is a neuroprotectant in the central nervous system

**DOI:** 10.3389/fncel.2015.00304

**Published:** 2015-08-17

**Authors:** Manuel Yepes

**Affiliations:** Department of Neurology and Center for Neurodegenerative Disease, Emory University School of Medicine and Veterans Affairs Medical CenterAtlanta, GA, USA

**Keywords:** cerebral ischemia, neurovascular unit (NVU), middle cerebral artery occlusion (MCAo), tissue-type plasminogen activator (tPA), neuroprotection, excitotoxicity, plasminogen

## Abstract

Tissue-type plasminogen activator (tPA) is a serine proteinase found not only in the intravascular space but also in a well-defined sub-set of neurons in the brain. tPA is rapidly released from neurons after either exposure to hypoxia or hypoglycemia *in vitro*, or the induction of cerebral ischemia *in vivo*. It has been proposed that tPA has a neurotoxic effect in the ischemic brain. However, recent evidence indicate that once released into the synaptic cleft tPA activates specific cell signaling pathways that promote the detection and adaptation to metabolic stress. More specifically, the non-proteolytic interaction of tPA with N-methyl-D-aspartate receptors (NMDARs) and a member of the low-density lipoprotein receptor (LDLR) family in dendritic spines activates the mammalian target of rapamycin (mTOR) pathway that adapts cellular processes to the availability of energy and metabolic resources. TPA-induced mTOR activation in neurons leads to hypoxia-inducible factor 1α (HIF-1α) accumulation, HIF-1α-induced expression and membrane recruitment of the neuronal transporter of glucose GLUT3, and GLUT3-mediated uptake of glucose. These and other data discussed in this Review suggest that the postulated neurotoxic effect of tPA needs to be reconsidered and instead indicate the emergence of a new paradigm: that tPA is an endogenous neuroprotectant in the central nervous system (CNS).

## Introduction

Tissue-type plasminogen activator (tPA) is a serine proteinase of 527 residues. It has an A chain with a finger, an EGF and two kringle domains, and a B chain with a protease domain. tPA is secreted as a single chain molecule (sctPA), but cleavage by plasmin at its Arg275-Ile276 peptide bond generates two-chain tPA (tctPA). sctPA is not a zymogen and in the presence of fibrin is almost as active as tctPA (Rijken et al., 1982).

Our understanding of tPA’s function in the brain has evolved rapidly. Indeed, although it was initially believed that endothelial cells (ECs) were the sole source of tPA and that its unique role was to catalyze the conversion of plasminogen into plasmin in the intravascular space, today we know that tPA is expressed in the brain parenchyma, where it has several functions, many of them independent of its ability to catalyze the conversion of plasminogen into plasmin.

## tPA in the Brain

To better understand tPA’s effect on neuronal survival is pivotal to consider its concentration in the brain parenchyma and to study its expression in the context of the neurovascular unit (NVU).

### Concentrations of tPA

The concentration of tPA in the intravascular space is 5 μM/L which correspond to approximately 70 pM (Nicoloso et al., 1988). In contrast, tPA’s concentration in the extracellular space of the brain is more difficult to quantify. However, taking into consideration the volume of brain tissue supplied by the middle cerebral artery (MCA) in the C57BL/6 mouse, the release of tPA from neuronal cultures (Echeverry et al., 2010), the area of the synaptic cleft, the concentration of tPA in the brain tissue under ischemic conditions and following the intravenous (IV) administration of recombinant tPA (rtPA; Haile et al., 2012), and a 60% contraction of the interstitial space in the ischemic brain (Hansen and Olsen, 1980), the extracellular concentration of tPA in the brain 3 h after MCA occlusion (MCAo) and IV treatment with either saline solution or 0.9 mg/Kg of rtPA is not superior to 1 nM and 2–4.5 nM, respectively. Although in all likelihood this is an overestimation and many other factors need to be considered to improve the accuracy of these calculations, it is clear that an experimental design with concentrations of tPA superior to 5 nM does not resemble tPA’s concentrations in an *in vivo* system, even after the administration of rtPA. This point is of key importance for the translatability to the clinic of results obtained in basic sciences laboratories dedicated to the study of tPA’s effect on neuronal survival.

### tPA in the Neurovascular Unit (NVU)

The NVU is a dynamic structure assembled by ECs surrounded by a basement membrane (BM) encased by perivascular astrocytes (PVA). Synapses are found approximately 13 μm away from the ECs (Zhang et al., 2005). This concept is highly relevant because following its IV administration rtPA permeates the brain parenchyma (Haile et al., 2012) potentially entering in contact with the synaptic space. Although microglia and pericytes should also be considered part of the NVU, a discussion of tPA’s expression and function in these cells is beyond the scope of this Review.

#### tPA in Endothelial Cells

Early studies in the non-human primate brain detected tPA antigen in a small fraction of ECs of the microvasculature, 90% of them pre-capillary arterioles and post-capillary veins (Levin and del Zoppo, 1994). Despite the importance of these findings, since the publication of this report more than 20 years ago, no further attempts have been made to study with new antibodies the expression of tPA in the brain vasculature. Nevertheless, it has been proposed that PVA regulate the expression of tPA mRNA in ECs (Tran et al., 1998) and that a number of stimuli including membrane depolarization induce its release into the intravascular space.

#### tPA in the Basement Membrane-Astrocyte (BM-A) Interface

tPA is abundantly expressed in PVA and its release into the BM-A interface has multiple effects including increase in the permeability of the blood-brain barrier (BBB; Yepes et al., 2003), detachment of astrocytic end-feet processes from the BM (Polavarapu et al., 2007), and induction of an NF-κB-regulated pro-inflammatory response (Zhang et al., 2007).

#### Neuronal tPA

Neurons are a major source of tPA synthesis *in vivo*. Studies using *in situ* zymography assays (Sappino et al., 1993) have detected tPA-catalyzed proteolysis in well-defined areas of the adult brain, namely hippocampus, hypothalamus, thalamus, amygdala, cerebellum and meningeal blood vessels. Intriguingly, tPA mRNA is found in several areas of the brain that do not display tPA-catalyzed enzymatic activity. That is the case of the cerebral cortex and the hippocampus. In the latter structure although tPA mRNA is widely expressed throughout all cell layers, only the CA2 and CA3 layers and the dentate gyrus (DG) show tPA-catalyzed proteolysis. It has been postulated that this discrepancy between tPA catalyzed-proteolysis and tPA mRNA expression may be due either to the localized expression of tPA inhibitors in those areas where tPA-catalyzed proteolysis is not observed, or to the accumulation of tPA protein at distant places of its synthesis, or to posttranslational regulation of tPA expression, or to the possibility that in some areas of the brain tPA has functions that do not require the generation of plasmin. Independently of the reason for this divergence, a substantial body of experimental evidence indicates that tPA is released at the neuronal growth cone (Krystosek and Seeds, 1981) and mediates neuronal migration (Seeds et al., 1999) and neurite outgrowth and remodeling during development (Lee et al., 2014) and in the ischemic brain (Shen et al., 2011). Likewise, tPA promotes the development of synaptic plasticity in *in vitro* and *in vivo* models of long-term potentiation (Qian et al., 1993), learning (Seeds et al., 1995, 2003), stress-induced anxiety (Pawlak et al., 2003), and visual cortex plasticity (Müller and Griesinger, 1998). Moreover, it has also been proposed that tPA plays a role on axonal remodeling after stroke (Liu et al., 2012).

## The Neurotoxic Effect of tPA

### History

One of the first achievements in our understanding of tPA’s role in the brain was provided by the observation that it is induced as an immediate-early gene during seizures and long-term potentiation, and to the identification of a functional link between tPA and N-methyl-D-aspartate receptors (NMDAR; Qian et al., 1993). Shortly thereafter it was found that membrane depolarization induces the rapid release of neuronal tPA by a mechanism that does not involve tPA mRNA or protein synthesis but requires the influx of Ca^+2^ (Gualandris et al., 1996). It was quickly recognized that the conditions associated with the release of neuronal tPA (i.e., membrane depolarization, increase in the intracellular concentrations of Ca^+2^ and NMDAR activation) also underlie the pathophysiological events observed in several neurological diseases such as cerebral ischemia, head trauma and seizures. This led to study in animal models of experimental cerebral ischemia changes in tPA activity in the brain tissue following MCAo. Although the interpretation of the initial studies was confusing, soon was evident that tPA activity increases rapidly in the ischemic tissue after MCAo (Wang et al., 1998; Yepes et al., 2003), and that this surge disappears at later time points when plasminogen activator inhibitor-1 (PAI-1) antigen is detected (Hosomi et al., 2001).

These reports were rapidly followed by the seminal observation that mice genetically deficient in tPA (tPA^−/−^) in all the cellular components of the NVU have a ~41% decrease in the volume of the ischemic lesion following MCAo (Wang et al., 1998; Nagai et al., 1999). The impact of these findings was contrasted by a later report from a different group of investigators using a similar experimental design that found just the opposite: an increase in the volume of the ischemic lesion in tPA^−/−^ mice (Tabrizi et al., 1999). Shortly thereafter a group of researchers discovered neuroserpin (Osterwalder et al., 1996), an axonally secreted serine proteinase inhibitor preferentially expressed in neurons (Hastings et al., 1997). This finding was followed by biochemical studies that revealed that although neuroserpin is an efficient inhibitor of plasminogen activators and plasmin, it has a higher affinity for tctPA (*k_i_*: 6.2 × 10^5^ M^−1^ S^−1^), than either sctPA (*k*_i_: 8.0 × 10^4^ M^−1^ S^−1^), or high molecular weight uPA (*k*_i_: 2.5 × 10^4^ M^−1^ S^−1^), or low molecular weight uPA (*k*_i_: 9.2 × 10^3^ M^−1^ S^−1^), or plasmin (*k*_i_: 3.6 × 10^2^ M^−1^ S^−1^; Hastings et al., 1997). These observations led to postulate neuroserpin as the inhibitor of tPA in the brain (Yepes and Lawrence, 2004). With this premise in mind, later studies with animal models of ischemic stroke revealed that either treatment with recombinant neuroserpin (Yepes et al., 2000) or genetic overexpression of neuroserpin (Cinelli et al., 2001) decreases the volume of the ischemic lesion following MCAo. Despite the discrepancies between animal studies with tPA^−/−^ mice, these results helped to advance the concept that tPA has a neurotoxic effect in the ischemic brain. Since then several research groups have reported potential mechanisms for tPA’s harmful effects and some have attempted to develop therapeutic strategies to antagonize it.

### Clinical Relevance

The idea borne from basic sciences laboratories that tPA has a neurotoxic effect in the ischemic brain was in striking opposition to an almost simultaneous publication by the National Institute of Neurological Disorders and Stroke (NINDS) of a clinical study indicating that IV treatment with rtPA leads to complete or nearly complete neurological recovery in a significant number of acute ischemic stroke patients (The National Institute of Neurological Disorders and Stroke rt-PA Stroke Study Group, 1995). Notably, although since then several clinical studies have shown that besides improving neurological outcome rtPA also increases the risk of intracerebral hemorrhage (Hacke et al., 2004) and augments the permeability of the BBB (Kidwell et al., 2008), to this date and after more than 100,000 rtPA-treated acute ischemic stroke patients, no clinical study has shown a neurotoxic effect for rtPA. The translational impact of this disagreement between basic and clinical research has been heightened by the observation that following its IV administration rtPA crosses the BBB and permeates the ischemic tissue (Haile et al., 2012). In conclusion, while basic researchers suggest that clinicians are treating acute ischemic stroke patients with a neurotoxic agent, clinicians are developing protocols to increase the number of rtPA-treated patients. This discrepancy needs to be resolved promptly because it has questioned the translatability into the clinic of tPA’s basic research.

### A New Look at Old Data

The translational importance of the findings described above warrants a second examination of the published data with the help of information that was not available when the original results were published.

#### Does Genetic Deficiency of tPA Decrease the Volume of the Ischemic Lesion after Experimental Induction of Ischemic Stroke?

There is a growing awareness among basic science stroke researchers that a large number of factors besides the actual MCAo influence the quantification of the volume of the ischemic lesion in experimental models of ischemic stroke (Sena et al., 2010). With this in mind, it is not surprising to find that while some studies have reported a decrease (Wang et al., 1998), others have found an increase (Tabrizi et al., 1999) in the volume of the ischemic lesion in tPA^−/−^ mice following MCAo. A similar reasoning applies to studies reporting either a deleterious (Wang et al., 1998), or beneficial (Wu et al., 2012), or lack of effect (Klein et al., 1999) on the volume of the ischemic lesion in animals treated with rtPA after MCAo.

In the analysis of these reports is important to consider that the earlier studies (Wang et al., 1998; Tabrizi et al., 1999) were criticized by their use of mice with two different genetic backgrounds: C57BL/6 and SV129. However, the validity of these concerns is difficult to evaluate because although it was initially believed that these two strains of mice have different vulnerability to the harmful effects of cerebral ischemia and excitotoxicity (Carmichael, 2005), later studies using magnetic resonance imaging (MRI) indicated that they are equally susceptible to both forms of injury (Pham et al., 2010). Thus it is problematic to invoke this factor to explain the opposite results observed in these studies. It also should be kept in mind that although the currently available tPA^−/−^ mice lack the protease domain they still harbor tPA’s finger, EFG and kringle domains. This fact has gained significant relevance over the last 10 years with the identification of several functions of tPA in the brain that do not require the conversion of plasminogen into plasmin. Therefore, it is difficult to assure that the finger and kringle domains do not have an effect in the final outcome of the ischemic injury in tPA^−/−^ mice.

It is also relevant to consider that tPA^−/−^ mice are deficient on tPA in all the cellular components of the NVU. Because tPA plays an unique and different role in ECs, PVA and neurons, it is difficult to determine whether the final outcome observed in tPA^−/−^ mice after MCAo is due to the effect of the lack of tPA on either the permeability of the BBB, or neuronal survival, or microglial activation. These are highly dynamic and variable processes that need to be dissected to have a better understanding of tPA’s role in the ischemic brain. A step to address this problem was recently taken by a group of investigators that reported that mice overexpressing tPA only in neurons have ~40% decrease in the volume of the ischemic lesion following MCAo (Wu et al., 2012). Based on these data and our knowledge that tPA increases the permeability of the BBB in rodents (Yepes et al., 2003) and humans (Kidwell et al., 2008), is plausible to postulate that the decrease in the volume of the ischemic lesion following MCAo observed by some investigators in tPA^−/−^ mice may be due to abrogation tPA-induced increase in the permeability of the BBB instead of a direct effect on neuronal survival.

The data obtained from mice overexpressing neuroserpin and rats treated with recombinant neuroserpin after MCAo also deserves careful attention. Certainly, the observation of unchanged tPA activity in animals genetically deficient in neuroserpin (Madani et al., 2003) and the report that neuroserpin also decreases the volume of the ischemic lesion in tPA^−/−^ mice (Wu et al., 2010), argue against tPA inhibition as a mechanism whereby neuroserpin decreases the volume of the ischemic lesion in these animals.

In summary, a new look at the available data suggests that the determination of the volume of the ischemic lesion in tPA^−/−^ mice or in animals either overexpressing neuroserpin or treated with recombinant neuroserpin after MCAo may not be sufficient evidence to conclude that tPA has a neurotoxic effect in the ischemic brain. Instead, data obtained from studies using animals overexpressing tPA only in neurons suggest that tPA may have a neuroprotective effect.

#### Does tPA Induce Neuronal Death?

A strategy to examine the effect of tPA on neuronal survival without the confounding effects of tPA derived from other cellular components of the NVU is to study cell death in neuronal cultures incubated with rtPA. Data obtained with this experimental design unambiguously indicate that rtPA, even at very high concentrations, does not induce neuronal death (Wu et al., 2013). To reconcile these observations with the harmful effect of tPA reported by some studies with tPA^−/−^ mice subjected to MCAo, some investigators postulated the hypothesis that while tPA does not directly cause neuronal death, it could act as a mediator of the deleterious effects of other agents known to cause neuronal death in the ischemic brain. This hypothesis is supported by studies performed with an animal model of neurodegeneration, namely the nervous (nr) mutant mouse, indicating that Purkinje cell degeneration correlates with high levels of tPA activity in the cerebellum (Li et al., 2006, 2013), and that mice overexpressing tPA in neurons develop Purkinje cell damage and ataxia (Cops et al., 2013). Although these studies do not indicate that tPA directly induces neuronal death, they suggest that under certain conditions tPA may facilitate the neurotoxic effect of other agents. Because the excitotoxic release of neurotransmitters underlies neuronal death in both, the ischemic brain and neurodegenerative diseases, then the obvious question was whether tPA mediates excitotoxin-induced neuronal death.

#### Does tPA Mediate Excitotoxin-Induced Neuronal Death?

The term excitotoxicity was coined in 1969 to describe the harmful effect on neuronal survival caused by prolonged and excessive activation of receptors of excitatory neurotransmitters (Olney, 1969). Since then it has been demonstrated that glutamate-induced excitotoxicity activates signaling pathways that lead to neuronal death in several neurological diseases including cerebral ischemia, seizures and head trauma (Choi, 1988). To study whether tPA mediates excitotoxin-induced neuronal death a group of researchers quantified neuronal demise in the hippocampus of tPA^−/−^ mice injected into the CA1 layer with kainic acid (KA), an excitatotoxic glutamate analog. Using this experimental paradigm these investigators reported extensive neuronal loss in the CA1 region of Wt but not tPA^−/−^ mice (Tsirka et al., 1995), and subsequent studies led them to propose that this effect was mediated by plasmin-induced proteolysis of laminin in the extracellular matrix (Chen and Strickland, 1997). These studies originated the idea that although under non-ischemic conditions tPA is pivotal for neurophysiological processes required for normal brain function such as neurite outgrowth (Krystosek and Seeds, 1981), learning (Seeds et al., 2003) and memory (Baranes et al., 1998), during cerebral ischemia it plays a completely opposite role as a mediator of excitotoxin-induced neuronal death.

In the murine hippocampus tPA protein is abundantly expressed in the CA2 and CA3 but not the CA1 layer (Salles and Strickland, 2002), which is known to have a high vulnerability to KA-induced cell death (Tsirka et al., 1995). This concept acquired special relevance when the same group of investigators found that the injection of KA causes a transient increase in tPA activity in some cells of the CA1 layer (Salles and Strickland, 2002). The striking observation was that those cells that exhibited an increase in tPA activity also survived the excitotoxic injury. Thus, when analyzed together with a previous report by a different research group (Kim et al., 1999) indicating that tPA protects hippocampal cells from the harmful effects of KA, it is clear that a casual link between tPA and excitotoxin-induced neuronal death needs was not clear yet. Importantly, this group of investigators also observed tPA^−/−^ mice are resistant to KA-induced seizures (Tsirka et al., 1995). To analyze these observations is important to keep in mind that the onset and spreading of seizures throughout the brain requires the development of structural and functional changes in the synapse that allow the spread of the abnormal electrical activity throughout neuronal circuits. Thus, the higher resistance to pharmacologically-induced seizures in tPA^−/−^ mice could also be explained by the lack of tPA-induced synaptic changes needed for the onset and spread of a seizure.

To gain perspective for the analysis of these data is important to keep in mind that the intrahipocampal injection of KA also induces prolonged seizures, that although very often remain clinically unrecognized, cause widespread hippocampal cell death, particularly in the CA1 layer (Meldrum, 2002). Thus, with this experimental paradigm is difficult to analyze whether tPA mediates KA- or seizures-induced neuronal death. To answer this question, a research group used two different experimental approaches. In the first, they studied the clinical and electrophysiological spread of seizures throughout the limbic system in tPA^−/−^ and Wt mice injected with KA into the amygdala (Yepes et al., 2002). The fact that KA was not injected directly into the hippocampus allowed these researchers to dissect KA- from seizures-induced hippocampal neuronal death. These experiments indicated that genetic deficiency of tPA is associated with slower progression of KA-induced seizure activity throughout the limbic system and a decrease in seizures-induced hippocampal cell death. In the second approach, they used an animal model in which transient occlusion of both common carotid arteries causes hippocampal cell death (Echeverry et al., 2010). With this paradigm these investigators were able to evaluate the effect on neuronal survival of endogenous glutamate released from hippocampal neurons in response to an ischemic insult. These studies showed that, as it was demonstrated years before in animals treated with KA (Salles and Strickland, 2002), hippocampal ischemia also causes an increase in tPA activity in the hippocampal CA1 layer. Remarkably, these investigators found that, as was also reported by the earlier studies with KA-injected mice, CA1 neurons that exhibit an increase in tPA activity also survive the ischemic injury. In agreement with these observations, it was found that hippocampal neurons of tPA^−/−^ mice are more vulnerable to the ischemic injury than those of their Wt littermate controls. Together, these data suggested a neuroprotective role of tPA against ischemia-induced excitotoxic neuronal death.

To study the effect of tPA on excitotoxin-induced neuronal death without the confounding effect of seizures, a group of researchers injected NMDA, another glutamate analog, into the striatum followed by IV treatment with rtPA. The advantage of this model is that the injection of NMDA into the striatum causes neuronal death but not seizures. Using this approach, these investigators (Reddrop et al., 2005) found that 10 mg/Kg/IV of rtPA increases the volume of the necrotic lesion caused by the injection of NMDA. Moreover, the same group of investigators measured the volume of the necrotic area induced by the direct intrastriatal injection of NMDA alone or in the presence of 3 μg of either rtPA or desmoteplase, the plasminogen activator from the vampire bat *Demodus rotundus* (Liberatore et al., 2003), and found that tPA but not desmoteplase increased NMDA-induced neuronal death.

Together, these studies were interpreted as another demonstration that tPA enhances excitotoxin-induced neuronal death. Intriguingly, the dose of tPA used for the intracerebral injections was unusually large (3 μg) and at this moment is difficult to elucidate why desmoteplase did not potentiate NMDA’s effect. More importantly, the dose of IV rtPA administered by these researchers is widely used by many investigators because it is believed that rodent plasminogen is 10-fold more resistant than human plasminogen to the catalytic action of rtPA. However, this concept has been challenged, and later studies with animal models of embolic stroke indicate that the dose of rtPA used to treat acute ischemic stroke patients (0.9 mg/Kg/IV) is also effective in rodents (Haelewyn et al., 2010). This has proved to be a very important fact because a different group of investigators using the same experimental paradigm demonstrated that treatment with 0.9 mg/Kg/IV of rtPA has just the opposite effect: a decrease in the volume of the necrotic lesion induced by the intrastraiatal injection of NMDA (Wu et al., 2013). In line with these observations, the damage induced by the intrastrial injection of NMDA was significantly attenuated in mice overexpressing tPA in neurons.

These studies underscored the need to investigate the effect of different doses of tPA on excitotoxin-induced neuronal death. With this concept in mind, this group of investigators quantified neuronal survival in Wt cerebral cortical neurons incubated with NMDA in the presence of 0–500 nM of either proteolytically active tPA or a mutant of tPA with an alanine for serine substitution at the active site Ser481 that renders it unable to catalyze the conversion of plasminogen into plasmin (proteolytically inactive tPA; Wu et al., 2013). These experiments indicated that tPA causes a modest increase in NMDA-induced neuronal death only at doses greater than 100 nM which, as it was discussed above, result in a concentration of tPA not found in an *in vivo* system, even after treatment with rtPA. In sharp contrast with these findings, the most important observation was that at concentrations found in the ischemic brain, tPA attenuates NMDA-induced neuronal death by a mechanism that does not entail plasmin generation but requires a co-receptor function by a member of the low-density lipoprotein receptor (LDLR) family, most likely LRP1.

#### What is the Nature of tPA’s Interaction with NMDA Receptors (NMDARs)?

Despite abundant experimental evidence indicating that NMDARs mediate excitotoxin-induced neuronal death, clinical studies with ischemic stroke patients treated with NMDAR blockers were disappointing, showing either lack of or, in some cases, harmful effects that precluded their use (Davis et al., 2000). To understand this divergence between basic and clinical research is important to take into account that NMDARs are assembled by obligatory NR1 sub-units that interact with NR2A—D sub-units, generating a specific sub-unit composition that determines the function of the receptor. Hence, while NR2A-containing NMDARs are located in the synapse and their activation has been coupled to neuronal survival (Liu et al., 2007), most of the NR2B- and NR2D-containing NMDARs are extrasynaptic and linked to the activation of cell-death pathways (Hardingham and Bading, 2010).

Although a functional association between tPA and NMDARs has been identified by several researchers, it was not until 2001 when a group of investigators reported that at ~280 nM of tPA enhances NMDA-induced neuronal death via a plasminogen-independent proteolytic cleavage of the NR1 sub-unit of the NMDAR that leads to increase in Ca^+2^ permeability (Nicole et al., 2001). These striking results led to postulate the NR1 sub-unit as a therapeutic target to antagonize the purported deleterious effect of tPA on excitotoxin-induced neuronal death. Unfortunately, since then a number of investigators have been unable to replicate these findings (Matys and Strickland, 2003; Samson et al., 2008; Wu et al., 2013). The controversy has been further enhanced by an elegant study indicating that the interaction between tPA and NMDARs does not involve the cleavage of the NR1 sub-unit but instead that it requires a co-receptor function of a member of the LDLR family (Samson et al., 2008). Additionally, several investigators have convincingly demonstrated that NMDARs are a discrete proteolytic target for plasmin but not for tPA (Matys and Strickland, 2003; Yuan et al., 2009; Echeverry et al., 2010).

A further advance in our understanding of tPA’s interaction with the NMDAR was provided by subsequent studies indicating that at concentrations likely to be found in an *in vivo* system tPA protects neurons against excitotoxin-induced cell death (Wu et al., 2013). Furthermore, the same group demonstrated that 1–10 nM of tPA induces phosphorylation but not cleavage of the NR2A sub-unit of the NMDAR at Y1325, and that this event leads to ERK 1/2-mediated activation of the cAMP response element binding protein (CREB) and induction of the neuroprotective effect of the CREB-regulated activating transcription factor 3 (Atf3). In line with these observations Atf3’s down-regulation abrogated tPA’s protective effect against excitotoxin-induced neuronal death. In this study tPA induced phosphorylation, but not cleavage, of extrasynaptic NR2B sub-units at Tyr1472 only at concentrations greater than 200 nM. These findings were also observed in *in vivo* studies with mice overexpressing neuronal tPA. This work also indicated that the non-proteolytic interaction between tPA and NMDARs requires the co-receptor function of a member of the LDLR family. An important point brought to light by these studies is the existence of a dose-dependent effect of tPA on NMDAR phosphorylation, where a protective NR2A-mediated response is activated by concentrations of tPA expected in an *in vivo* system, while a harmful NR2B-mediated neurotoxic pathway is triggered by concentrations of tPA unlikely to be found in the synapse.

In summary, a link between tPA and excitotoxin-induced neuronal death is not clear yet. Furthermore, although the functional association between tPA and NMDARs has been substantiated by a large number of studies, the mechanism underlying this interaction and its impact on cell survival remain contentious. The resolution of this controversy if of the utmost importance not only for the clinical implications of these findings but also for their obvious repercussions on the conceptual advancement of the field of tPA research.

## The Emergence of a New Paradigm: tPA is a Neuroprotectant in the Central Nervous System (CNS)

Several lines of evidence suggest that tPA could instead be a neuroprotectant in the central nervous system (CNS). This concept, that was already suggested by earlier studies indicating that tPA protects neurons from excitotoxin-, seizures- (Kim et al., 1999) and serum deprivation-induced cell death (Liot et al., 2006), has been further advanced by more recent work with lower doses of rtPA and mice overexpressing tPA only in neurons.

### tPA Promotes Survival in Neurons Exposed to Oxygen and Glucose Deprivation (OGD)

To study tPA’s effect on neuronal survival we designed an *in vitro* model of ischemic stroke in which we quantified cell survival in Wt cerebral cortical neurons treated with 5 nM of rtPA between 5 min and 6 h after exposure to 55 min of oxygen and glucose deprivation (OGD). Our data indicated that treatment with rtPA protects cerebral cortical neurons from OGD-induced cell death. This effect is independent of tPA’s proteolytic activity and although is abrogated by NMDAR and LRP1 antagonism with MK-801 and the receptor-associated protein (RAP), respectively (Echeverry et al., 2010), it remains unchanged by inhibition of the tyrosine kinase receptor B (TrkB), the cognate receptor of brain-derived neurotrophic factor (BDNF). Intriguingly, the detection of a maximal neuroprotective effect in cells treated within the first 3 h after OGD, and still present albeit with less intensity in cells treated 6 h after the hypoxic insult, has a notable resemblance with the probability of neurological recovery observed in acute ischemic stroke patients treated with rtPA (Hacke et al., 2004).

The obvious lack of a clot in our *in vitro* system indicated that a mechanism other than thrombolysis mediates tPA’s neuroprotective effect. To better characterize these results Wt mice underwent transient MCAo followed immediately after reperfusion by treatment with 0.9 mg/Kg/IV of rtPA or a comparable volume of saline solution, and quantification of the volume of the ischemic lesion 24 h later. We found that treatment with rtPA decreases the volume of the ischemic lesion. However, because MCAo was performed with a nylon suture instead of a clot, it was evident that, as suggested by our *in vitro* studies, the mechanism of this effect is independent of tPA’s ability to generate plasmin. This was further confirmed by our finding that treatment with rtPA also decreases the volume of the ischemic lesion in mice genetically deficient on plasminogen (Plg^−/−^). These data indicated that tPA has a neuroprotective effect in the ischemic brain that is not mediated by the generation of plasmin and instead requires the co-receptor function of the NMDAR and a member of the LDLR family.

### tPA-induced Neuroprotection: A Proteomics Approach

These results implied the activation of a neuroprotective cell signaling pathway by tPA. To test this hypothesis, we used liquid chromatography coupled to tandem mass spectrometry (LC-MS/MS) and quantitative analysis to identify protein changes in Wt cerebral cortical neurons incubated with 5 nm of either proteolytically active or inactive rtPA. We found that compared to vehicle (control)-treated neurons, treatment with rtPA causes a >50% change in abundance in 589 unique neuronal proteins, by a mechanism that does not involve plasmin generation. Further studies with the Ingenuity Pathway Analysis tool showed that most of these proteins belong either to the oxidative phosphorylation (72 out of 160 proteins) or the mammalian target of rapamycin pathways (mTOR; 53 of 201 proteins) pathways. In agreement with these results, Western blot analysis indicated that either treatment of Wt cerebral cortical neurons with rtPA, or the IV administration of rtPA after the onset of cerebral ischemia, induces mTOR activation. More importantly, mTOR inhibition with rapamycin abrogated tPA-induced neuroprotection *in vitro* in cerebral cortical neurons exposed to OGD conditions and *in vivo* in Wt mice subjected to MCAo (Wu et al., 2012). Together, these data indicated that tPA’s neuroprotective effect is mediated by its ability to activate the mTOR pathway.

### tPA Promotes the Detection and Adaptation to Metabolic Stress

Hypoxia-inducible factor-1α (HIF-1α) is a trascription factor that plays a central role in hypoxia sensing and adaptation. HIF-1α expression is regulated by mTOR, and its accumulation has a neuroprotective effect in the ischemic brain (Shi, 2009). We found that either incubation with 5 nM of rtPA or the IV administration of 0.9 mg/Kg of rtPA induces HIF-1α accumulation in cultured neurons and in the ischemic brain, respectively. Furthermore, shRNA-induced HIF-1α down-regulation abrogated the neuroprotective effect of rtPA in neurons exposed to OGD conditions (Wu et al., 2012). More importantly, we found that tPA-induced mTOR-mediated HIF-1α accumulation leads to the recruitment of the neuronal transporter of glucose GLU3 to the neuronal plasma membrane. In line with these observations, tPA induced the uptake of glucose both in Wt cerebral cortical neurons *in vitro* and the ischemic brain *in vivo*.

## Proposed Mechanistic Model for tPA-Induced Neuroprotection

**Figure 1:**
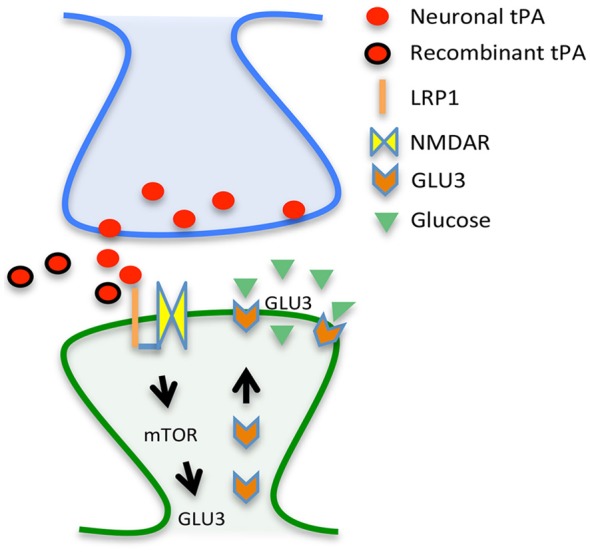
**Proposed mechanism for tPA-induced neuroprotection.** Tissue-type plasminogen activator (tPA) either released from the presynaptic terminal (red circles) or given as a treatment (rtPA; red circles with a black margin) interact with a co-receptor formed by the low density lipoprotein receptor-related protein (LRP1; orange line) and the NMDA receptor (yellow squares) on the surface of the dendritic spine (post-synaptic compartment), leading to mTOR activation and mTOR-induced synthesis of the glucose transporter GLU3 (orange arrows) with the resultant increase in the uptake of glucose (green triangles).

Based on these data and other results not discussed here (An et al., 2014), we propose a model whereby tPA either released in the synaptic cleft following the onset of cerebral ischemia or intravenously administered interacts with LRP1, leading to NMDAR-mediated mTOR activation, mTOR-induced HIF-1α accumulation, HIF-1α-induced recruitment of the neuronal transporter of glucose GLUT3 to the neuronal plasma membrane, and GLUT3-mediated uptake of glucose by neurons in the ischemic brain (Figure 1). In summary, here we propose a mechanistic model whereby tPA is a neuroprotectant in the ischemic brain by its ability to promote the detection and adaptation to the metabolic stress triggered by the lack of oxygen and glucose.

## Conflict of Interest Statement

The author declares that the research was conducted in the absence of any commercial or financial relationships that could be construed as a potential conflict of interest.
